# Three sympatric clusters of the malaria vector *Anopheles culicifacies* E (Diptera: Culicidae) detected in Sri Lanka

**DOI:** 10.1186/s13071-015-1286-3

**Published:** 2016-01-04

**Authors:** Iresha Nilmini Harischandra, Ranil Samantha Dassanayake, Bambaranda Gammacharige Don Nissanka Kolitha De Silva

**Affiliations:** Department of Zoology, Faculty of Applied Sciences, University of Sri Jayewardenepura, Gangodawila, Nugegoda, Sri Jayewardenepura, 10250 Sri Lanka; Department of Chemistry, Faculty of Science, University of Colombo, Cumarathunga Munidasa Mawatha, Colombo 04, 00300 Sri Lanka

**Keywords:** *Anopheles culicifacies* E, Microsatellite markers, Population genetic structure

## Abstract

**Background:**

The disease re-emergence threat from the major malaria vector in Sri Lanka, *Anopheles culicifacies,* is currently increasing. To predict malaria vector dynamics, knowledge of population genetics and gene flow is required, but this information is unavailable for Sri Lanka. This study was carried out to determine the population structure of *An. culicifacies* E in Sri Lanka.

**Methods:**

Eight microsatellite markers were used to examine *An. culicifacies* E collected from six sites in Sri Lanka during 2010-2012. Standard population genetic tests and analyses, genetic differentiation, Hardy-Weinberg equilibrium, linkage disequilibrium, Bayesian cluster analysis, AMOVA, SAMOVA and isolation-by-distance were conducted using five polymorphic loci.

**Results:**

Five microsatellite loci were highly polymorphic with high allelic richness. Hardy-Weinberg Equilibrium (HWE) was significantly rejected for four loci with positive *F*_IS_ values in the pooled population (*p* < 0.0100). Three loci showed high deviations in all sites except Kataragama, which was in agreement with HWE for all loci except one locus (*p* < 0.0016). Observed heterozygosity was less than the expected values for all sites except Kataragama, where reported negative *F*_IS_ values indicated a heterozygosity excess. Genetic differentiation was observed for all sampling site pairs and was not supported by the isolation by distance model. Bayesian clustering analysis identified the presence of three sympatric clusters (gene pools) in the studied population. Significant genetic differentiation was detected in cluster pairs with low gene flow and isolation by distance was not detected between clusters. Furthermore, the results suggested the presence of a barrier to gene flow that divided the populations into two parts with the central hill region of Sri Lanka as the dividing line.

**Conclusions:**

Three sympatric clusters were detected among *An. culicifacies* E specimens isolated in Sri Lanka. There was no effect of geographic distance on genetic differentiation and the central mountain ranges in Sri Lanka appeared to be a barrier to gene flow.

## Background

**Fig. 1:**
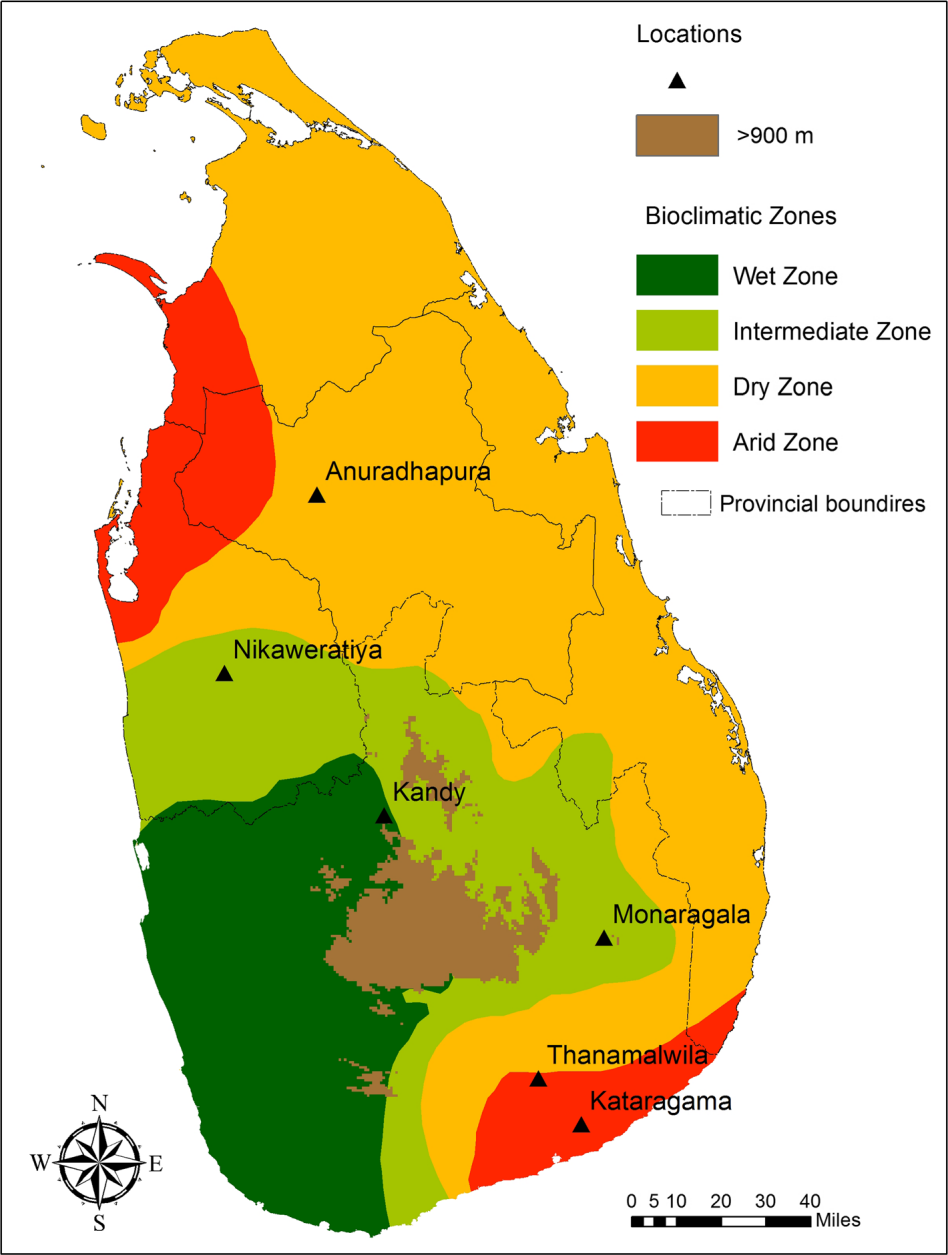
Map of Sri Lanka showing climatic zones and sample collection sites

*Anopheles culicifacies* Giles *sensu lato,* the major malaria vector in Sri Lanka, is widely distributed across the dry and intermediate zones of the country (Fig. 1). *An. culicifacies* is comprised of five morphologically indistinguishable sibling species that were reported in India and provisionally designated as A, B [1], C [2], D [3] and E [4]. Species B and E are found in Sri Lanka, where E is the major vector [5, 6] and B is the poor vector. Sympatrically distributed B and E species show variations in insecticide resistance, host feeding preference, longevity and Y-chromosome polymorphism [5, 6].

Until recently, *Plasmodium vivax* and *P. falciparum* parasite infections caused millions of clinical malaria cases in Sri Lanka that resulted in thousands of deaths [7]. Although malaria control measures in Sri Lanka have reduced the number of reported annual cases to several hundred, imported cases can still occur and thus may create a high risk for disease re-emergence [8]. The main malaria control method in Sri Lanka was vector controlling through residual insecticide spraying, which is now less frequent. However, recent research findings show that vector species can tolerate a variety of harsh environmental conditions including salinity and pollution [9, 10]. Thus, there is a potential for malaria to spread if a *Plasmodium* outbreak occurs.

The dynamics of malaria vector mosquito populations can be accurately predicted using analyses of population genetic structures and gene flow. Such knowledge would be useful for implementing new strategies to monitor malaria vectors as well as to understand disease epidemiology and the spread of insecticide resistance [11].

Microsatellites are highly polymorphic and evolve more rapidly than nuclear or mitochondrial DNA, and thus they are widely used for genetic analyses of different mosquito vectors such as *An. gambiae* [12, 13], *An. sinensis* [14], *An. arabiensis* [11, 15–17] and *An. funestus* [18]. In India, microsatellite markers have been isolated and the population genetic structure has analyzed for the *An. culicifacies* sibling species A [19, 20]. However, the population genetics of *An. culicifacies* in Sri Lanka have not been studied and only various genetic markers have been used to identify sibling species. Therefore, in this study, microsatellite markers developed to analyze the sibling species A in India [19] were used to evaluate the genetic structure of *An. culicifacies* E populations in Sri Lanka.

## Methods

### Mosquito samples

Wild engorged female *An. culicifacies* mosquitoes were collected between January 2010 and December 2012 from six different sites in Sri Lanka: Anuradhapura (8°21’N, 80°23’E), Kandy (7°17’N, 80°38’E), Nikaweratiya (7°43’N, 80°07’E), Thanamalwila (6°25’N, 81°07’E), Monaragala (6°54’N, 81°10’E) and Kataragama (6°40’N, 81°32’E) (Fig. 1). Multiple collections at each site were conducted. No collection sites were located in the Northern and Eastern parts of Sri Lanka due to infrequent indoor spraying (IRS) of insecticides arising from 30 years of civil war in these regions. Cytogenetically identified species E mosquitoes were used for microsatellite genotyping.

### DNA extraction and microsatellite genotyping

Genomic DNA was extracted from mosquitoes using a phenol:chloroform extraction method [21]. Out of 13 microsatellite loci used in the genetic analysis of species A and B in India [19], eight loci (AcAIIB5, AcAVB93, AcAVB93A, AcAVIB213, AcAVIIIB40, AcA36, AcA59, AcA75) were selected for this analysis based on PCR amplification of corresponding loci in sibling E. PCR was carried out as described previously [19] using forward primers that were labeled with HEX or FAM markers. The PCR products were genotyped (Macrogen Inc., South Korea) and allele scores determined according to the fragment size using Peak Scanner software (Applied Biosystems, USA). A total of 193 individuals were genotyped from six (6) sampling sites (*N* = 29, 33, 32, 36, 31, and 42 for Anuradhapura, Monaragala, Thanamalwila, Kandy, Kataragama and Nikaweratiya, respectively).

### Genetic analysis

The genetic analysis was carried out using five polymorphic loci: AcAIIB5, AcAVB93, AcAVIB213, AcA36 and AcA59. The genetic diversity within samples and overall was calculated for each locus by estimating allele frequencies, number of alleles, allele richness and in breeding coefficient (*F*_IS_) using FSTAT software v2.9.3.2 [22]. The presence of null alleles [23] at each locus was calculated with Micro-Checker 2.2.3 [24]. Genotypic frequencies were tested against the Hardy-Weinberg Equilibrium (HWE) within populations using Arlequin v3.1 [25]. An unbiased estimate of the *p*-value for each locus was then calculated by exact test using the Markov chain method [26], with a forecasted chain length of 1,000,000 steps and dememorization steps of 100,000. Pairwise linkage disequilibrium between all pairs of loci was calculated with Arlequin v3.1 and GENEPOP v4.3 [27]. All analyses were performed with 1,000 dememorizations, 100 batches and 1,000 iterations per batch.

To determine the population substructure, *F*_ST_ values were calculated followed by overall tests for differentiation using bootstrap-correlated Fisher’s exact tests in FSTAT. Wright’s F-statistics [28] in population pairs using Arlequin v3.1 and the Weir and Cockerham method [29] in FSTAT were used to evaluate the level of genetic differentiation between populations. Bonferroni corrections were performed for all tests that involved multiple comparisons.

The long term effective population size (*Ne*) was estimated [30] based on the expected heterozygosity at each microsatellite locus assuming a Stepwise Mutation Model (SMM) using the formula *Ne*μ = {[1/(1-*He*)]^2^-1}/8 [30, 31], where *H*_*e*_ is the expected heterozygosity under HWE and ‘μ’ is the microsatellite mutation rate. Proposed mutation rates for *An. gambiae* [32] were used taking into account that the average mutation rate varies little, even between well separated species [33]. *Ne* estimates were calculated in a relative scale, using the product of *Ne*μ as a proxy of *Ne* for each locality to avoid bias due to incorrect estimation of the mutation rate [33]. The effective migration rate between localities (*Nm* values) was estimated using pairwise *F*_ST_ values [34]. The analysis of molecular variance (AMOVA) in Arlequin v3.1 was used to examine genetic variation distributions. The isolation by distance model was used to test for any effect of distance on genetic differentiation. The significance of the regression of genetic differentiations on geographic distance between sample pairs was tested using the Mantel test [35] in the IBD software package [36], with 100,000 permutations and the regression of *F*_ST_ /(1-*F*_ST_) on the natural log (*ln*) of geographic distance [37]. Spatial analysis of molecular variance (SAMOVA) analysis was performed using SAMOVA software v1.0 [38] to investigate the spatial genetic structure and to identify any possible barriers to gene flow between collection sites.

Bayesian analysis was carried out to identify possible clusters (*K*) within the studied population using STRUCTURE 2.3.4 software [39]. Data sets were used without prior information for the sampling locations and assuming a model wherein allele frequencies were correlated within populations. The admixture model was used that allowed for some mixed ancestry within individuals and α was allowed to vary. Twenty independent runs were performed for each value of *K* (*K* =1 to 10) with a burn-in period of 100,000 iterations and 10,000 steps for Monte Carlo Markov Chain (MCMC) replications. The Evanno method [40] in the program STRUCTURE HARVESTER v0.6.8 [41] was used to determine the most likely number of clusters. This method includes an *ad hoc* quantity, D*K*, which is based on the second order rate of change of the likelihood function between successive values of *K*.

## Results

### Genetic diversity

Among all 5 loci, a total of 49 alleles were observed in the analysis. The number of alleles per locus ranged from 6 to 14. Locus AcA59 had the highest number of alleles among all populations (14) and the AcAIIB5 locus had the fewest (6). Locus AcA59 was also the most polymorphic, with allele frequencies ranging from 0.19 to 0.36, and a maximum value at all test sites. Allele richness based on a minimum sample size of 26 diploid individuals per locus was between 4.963 and 9.830, with locus AcA59 showing the highest richness for all sites (Table 1).

**Table 1:** Genetic diversity at microsatellite loci of sibling species E at six sampling sites

Locus	Locations							
		Anu	Mon	Tha	Kan	Kat	Nik	Pooled population
	N	29	33	32	26	31	42	193
AcA59	A	8	11	11	10	9	9	14
	*Rs*	7.896	10.686	10.432	10.000	8.829	7.901	9.830
	*F*_IS_	0.232*	0.246*	0.085*	0.303*	−0.040*	0.100*	0.177*
	*r*	0.1204	0.1311	0.0356	0.1662	−0.0273	0.0458	0.1204
	*H*_*e*_	0.75983	0.88112	0.88641	0.82278	0.83818	0.76592	0.85555
	*H*_*o*_	0.58621	0.66667	0.81250	0.57692	0.87097	0.69048	0.70466
AcAVIB213	A	7	8	8	9	5	7	12
	*Rs*	6.688	7.695	7.582	9.000	4.835	6.282	7.785
	*F*_IS_	0.059*	0.202	0.072	0.332	0.048	−0.026	0.136*
	*r*	0.0213	0.1034	0.029	0.1865	0.0159	−0.0187	0.0213
	*H*_*e*_	0.62250	0.68159	0.57192	0.74359	0.47382	0.58032	0.62312
	*H*_*o*_	0.58621	0.54545	0.53125	0.50000	0.45161	0.59524	0.53886
AcAIIB5	A	4	4	5	5	2	5	6
	*Rs*	3.999	3.958	5.000	5.000	2.000	4.999	4.963
	*F*_IS_	−0.387*	0.660*	0.326*	0.201*	−0.176	0.387*	0.319*
	*r*	−0.1672	0.4824	0.1847	0.1001	−0.0877	0.2318	−0.1672
	*H*_*e*_	0.65094	0.61678	0.69196	0.76697	0.27499	0.77281	0.71499
	*H*_*o*_	0.89655	0.21212	0.46875	0.61538	0.32258	0.47619	0.48705
AcAVb93	A	5	5	6	7	4	6	9
	*Rs*	4.887	4.998	5.962	7.000	3.839	5.853	6.522
	*F*_IS_	0.345*	−0.285	0.120*	0.096*	−0.426	−0.008*	0.047*
	*r*	0.1972	−0.1302	0.0548	0.04	−0.1801	−0.0101	0.1972
	*H*_*e*_	0.62795	0.56830	0.67312	0.76471	0.56954	0.73207	0.70161
	*H*_*o*_	0.41379	0.72727	0.59375	0.69231	0.80645	0.73810	0.66839
AcA36	A	6	4	5	5	2	6	8
	*Rs*	5.990	4.000	4.807	5.000	2.000	5.217	5.664
	*F*_IS_	0.077	0.149	−0.369	0.086	−0.429	−0.071	−0.038
	*r*	0.0306	0.0718	−0.1606	0.0342	−0.181	−0.0398	0.0306
	*H*_*e*_	0.67151	0.67506	0.64286	0.67195	0.43205	0.46730	0.60402
	*H*_*o*_	0.62069	0.57576	0.87500	0.61538	0.61290	0.50000	0.62694
All Loci	A	6	6.4	7	7.2	4.4	6.6	9.8
	*Rs*	5.892	6.267	6.756	7.2	4.299	6.24	6.953
	*F*_IS_	0.070	0.206	0.054	0.207	−0.188	0.097	0.135
	*r*	0.0345	0.1002	0.0444	0.0825	0.0666	0.0233	0.0345
	*H*_*e*_ *avg*	0.66655	0.68457	0.69325	0.75400	0.51772	0.66368	0.69986
	*H*_*o*_ *avg*	0.62069	0.54545	0.65625	0.60000	0.61290	0.60000	0.60519

N- number of samples, A = number of alleles, *F*_IS_ – inbreeding coefficient, *Rs* – allele richness, *r*- null allele frequency, *H*_*e*_– expected heterozygosity, *H*_*o*_– observed heterozygosity, All loci, all samples – mean values over loci and populations. Probability test against HWE * *p* < 0.006 after Bonferroni correction for the pooled population and *p* < 0.001 for the sampling sites. Anu-Anuradhapura, Mon-Monaragala, Tha-Thanamalwila, Kan-Kandy, Kat-Kataragama, Nik-Nikaweratiya

### Hardy-Weinberg Equilibrium (HWE) and linkage disequilibrium

Hardy-Weinberg Equilibrium was significantly rejected for four loci: AcA59, AcAVIB213, AcAIIB5 and AcAVb93, with positive *F*_IS_ values in the pooled population (Table 1) after Bonferroni correction (*p* < 0.0100). A marked deviation in AcA59, AcAIIB5 and AcAVb93 was observed at the sampling sites. Locus AcA59 significantly deviated at all six sampling sites. Meanwhile, AcAIIB5 deviated at five sampling sites and confirmed HWE in Kataragama. The AcAVb93 locus deviated at all sites except for Kataragama and Monaragala. Kataragama specimens showed HWE for all loci except AcA59 (*p* < 0.0016). Observed heterozygosity was less than the expected heterozygosity at all test sites except for Kataragama, which had negative *F*_IS_ values that indicted a heterozygosity excess.

Two pairs of loci in the pooled population (AcAVb93 - AcA59 and AcAVb93 - AcAIIB5) were significant in the exact test for linkage disequilibrium (*p* < 0.005). Among the collection sites, only one pair of loci (AcAVb93 - AcA59) in Anuradhapura showed significant linkage disequilibrium.

### Genetic differentiation and isolation by distance

Genetic variability between populations was estimated using pairwise *F*_ST_ values. The genetic differentiation was significant in all 15 population pairs (*p* < 0.0033) (Table 2). Significant *F*_ST_ values ranged from 0.03428 to 0.20299. The shortest distance between populations was between Monaragala and Kataragama (22 km) and the longest distance was between Kataragama and Anuradhapura (239 km). The highest levels of genetic differentiation were observed between Kataragama and Anuradhapura (239 km), Kataragama and Kandy (124 km) and Kataragama and Nikaweratiya (198 km) (Table 2). Despite this range of distances, the observed significant genetic differentiation appears to be independent of geographic distance between populations.

**Table 2:** *F*_ST_ and *Nm* values for pairwise comparisons of *An. culicifacies* sibling species E

	Anu	Mon	Than	Kan	Kat	Nik
Anu	0	2.66	2.72	5.67	0.98	6.97
Mon	0.08592*	0	50.35	5.81	3.89	4.46
Than	0.08415*	0.00494*	0	7.01	6.40	5.81
Kan	0.04221*	0.04122*	0.03445*	0	1.76	7.04
Kat	0.20299*	0.06039*	0.03759*	0.12416*	0	1.85
Nik	0.03463*	0.05306*	0.04125*	0.03428*	0.11896*	0

*p < 0.0033 after Bonferroni correction, figures above diagonal are *Nm* values and below diagonal are *F*_ST_ (Slatkin linearized *F*_ST_ as t/M = *F*_ST_/(1-*F*_ST_). Anu-Anuradhapura, Mon-Monaragala, Tha-Thanamalwila, Kan-Kandy, Kat-Kataragama, Nik-Nikaweratiya

**Fig. 2:**
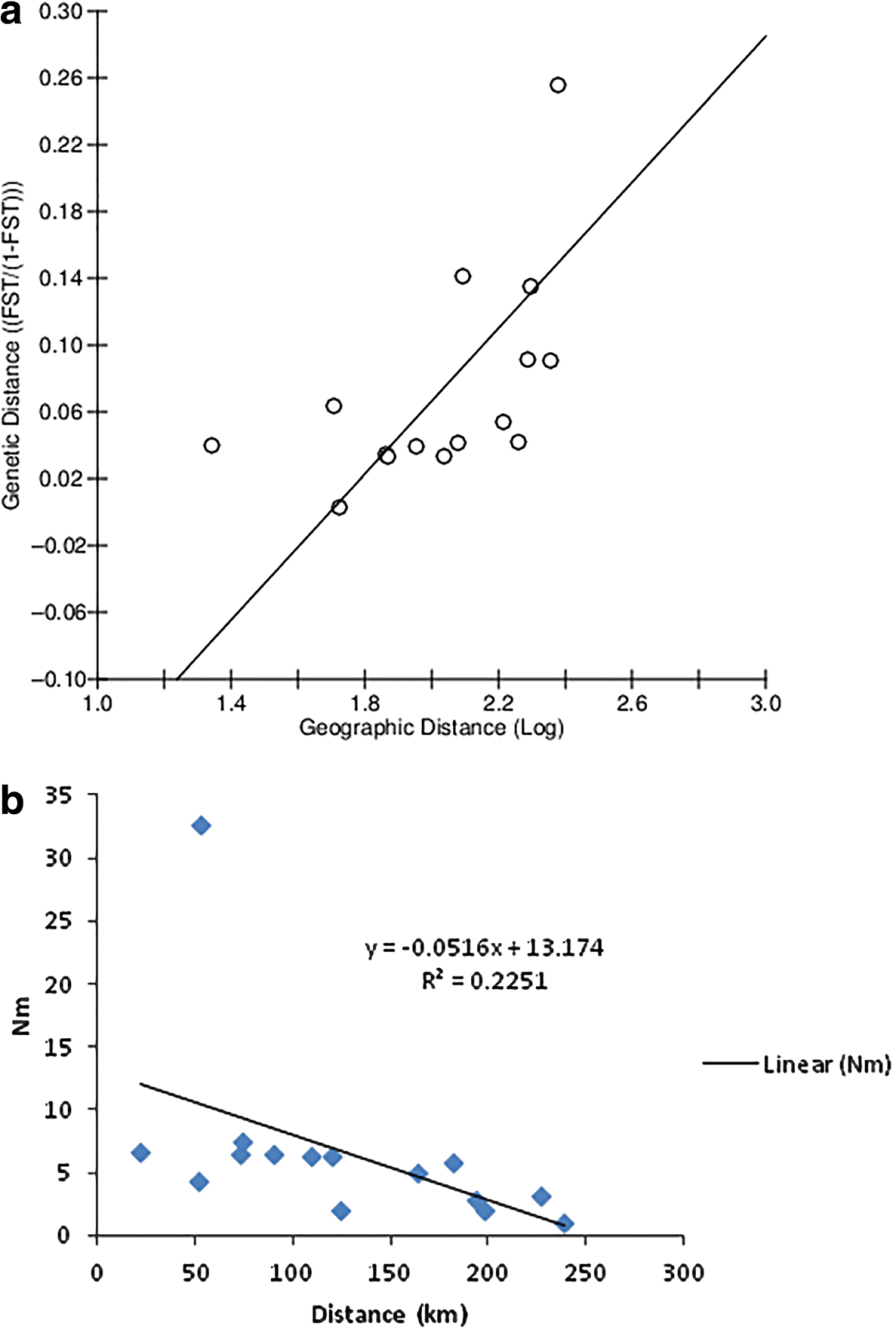
**a** Correlation between *F*_ST_/(1-*F*_ST_) of population pairs and geographical distances (km). **b** Correlation between population pair distances (km) and *Nm* values

The analysis of molecular variance (AMOVA), which is calculated based on *F*_ST_ values, showed greater variation within population pairs (86.44 %) than that among the populations (13.56 %), thus confirming genetic differentiation between population pairs. The Mantel test revealed that there is no significant correlation between pairwise genetic distance (*F*_ST_/(1- *F*_ST_) and the natural logarithm of pairwise geographical distance (r^2^ = 0.448, *p* = 0.0180), which supports the lack of correlation between the genetic differentiation of populations and geographic distances between population pairs (Fig. 2a).

### Gene flow

*Nm* values (number of migrants per population per generation) were calculated based on *F*_ST_ values as t/M = *F*_ST_/(1-*F*_ST_) for all population pairs (Table 2). The maximum differentiation (lowest *Nm* value) was observed between Kataragama and Anuradhapura populations (*Nm* = 0.98), Kataragama and Kandy populations (*Nm* = 1.76), and Kataragama and Nikaweratiya (*Nm* = 1.85) populations, which were separated by 239 km, 124 km and 198 km, respectively. The highest degree of genetic differentiation in terms of *F*_ST_ comparisons was also observed for these three population pairs, suggesting that a barrier to gene flow may exist within these populations. Monaragala and Thanamalwila, which are separated by 53 km, showed the highest *Nm* value (50.35) and the least genetic differentiation. According to the *Nm* values and the geographic distances between the populations, there is no correlation between the measure of gene flow (*Nm*) value and the distance (Fig. 2b), further supporting that the observed genetic differentiation is not related to geographic distance. Moreover, SAMOVA analyses based on the assumption of different numbers of population groups (2, 3, and 4 groups) showed no genetic differentiation among groups (Table 3), although there was a possible barrier to gene flow across Sri Lanka near the Kandy site that roughly separates the Anuradhapura and Nikaweratiya populations from the Monaragala, Thanamalwila and Kataragama collection sites (Fig. 4). Estimates of long term effective population sizes (*Ne*) ranged between 4,124 and 12,034 for collecting sites while 12,626 for the pooled population (Table 4). The lowest effective population size was reported from Kataragama (4,124).

**Table 3:** Results of SAMOVA. The most likely groupings of populations are presented assuming the presence of 2, 3, or 4 groups

Number of *K* groups	Structure tested	Variance among groups %	*F*_CT_	*p* value
2	Group 1 (Kat)	0.10180	0.05539	>0.05
	Group 2 (Anu, Mon, Tha, Kan, Nik)			
3	Group 1 (Anu, Kan, Nik)	0.09424	0.05252	>0.05
	Group 2 (Kat)			
	Group 3 (Mon, Tha)			
4	Group 1 (Kan)	0.09455	0.05302	>0.05
	Group 2 (Kat)			
	Group 3 (Mon, Tha)			
	Group 4 (Anu, Nik)			

Anu-Anuradhapura, Mon-Monaragala, Tha-Thanamalwila, Kan-Kandy, Kat-Kataragama, Nik-Nikaweratiya

**Table 4:** Effective population size (*Ne*) estimates based on genetic diversity (expected heterozygosity) at each collection site, assuming a stepwise mutation model

Location	*He*	*Ne*	RR
Anuradhapura	0.66655	9992	2.42
Monaragala	0.68457	11313	2.74
Thanamalwila	0.69325	12034	2.92
Kandy	0.75400	19405	4.70
Kataragama	0.51772	4124	1
Nikaweratiya	0.66368	9801	2.38
Pooled population	0.69986	12626	3.06

*He* - unbiased heterozygosity, *Ne -* mean effective population size calculated across all loci, μ - mutation rate, RR - relative ratio of *Ne*μ compared to the *An. culicifacies* population with the smallest effective population size (Kataragama)

### Bayesian clustering analysis of populations

**Fig. 3:**
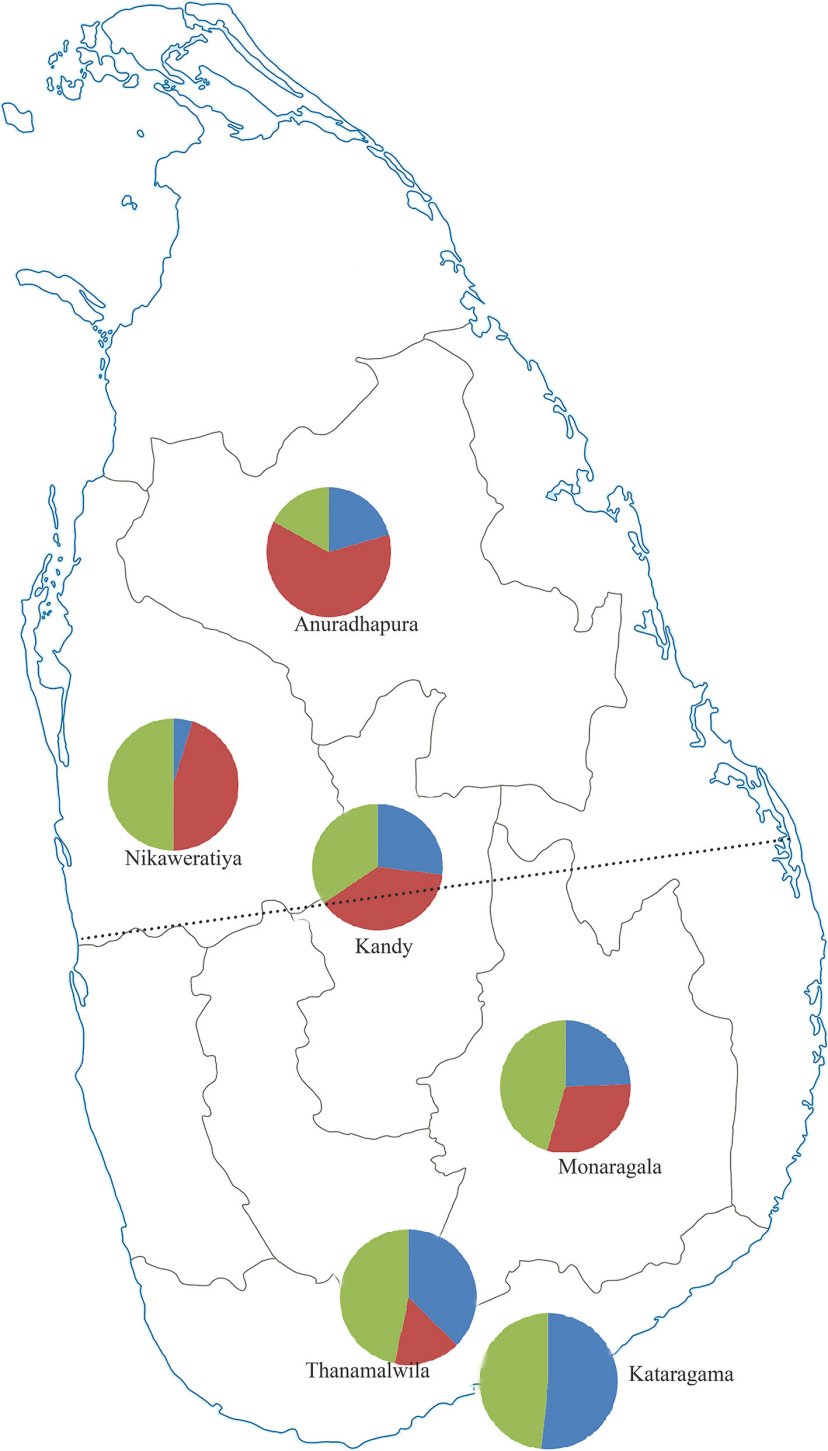
Graphical representation of cluster proportions at the six collection sites. The dotted line shows the barrier to gene flow (cluster I-red, cluster II-green, cluster III-blue)

**Fig. 4:**
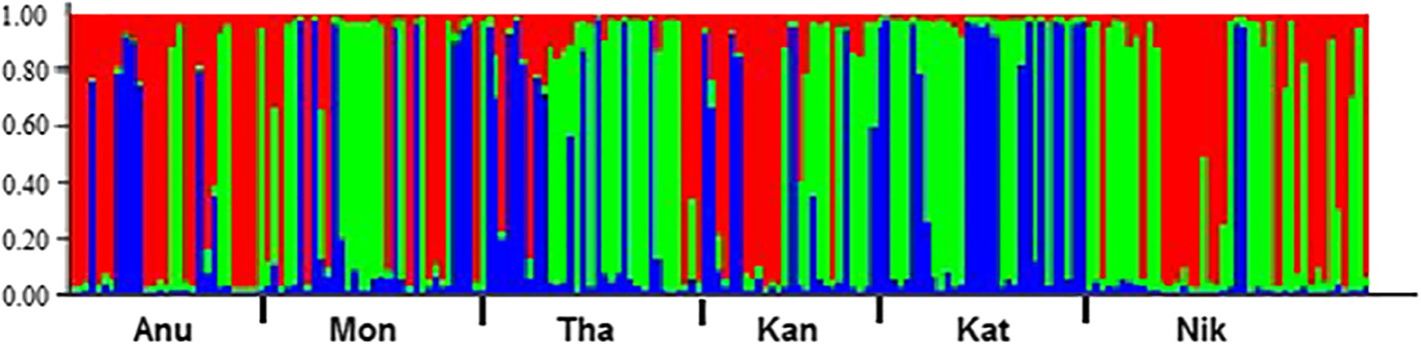
Bayesian cluster analysis using STRUCTURE. Graphical representation of the data set for the most likely *K* (*K* = 3). Each color corresponds to a suggested cluster and each individual is represented by a vertical bar. X-axis – population codes, Y-axis – probability of assignment of each cluster

The Bayesian cluster analysis divided the pooled population into three main clusters according to the genotypic variations (Posterior probability of Bayesian clustering Ln(D) likelihood score optimized for K = 3 clusters) (Fig. 3). Three clusters were mixed outputs of all sampled sites except for Kataragama, where individuals were assigned only to clusters II and III. Clusters I, II and III had 62, 80 and 51 individuals, respectively, and the percentages of each cluster at the sampling sites are shown in Fig. 4. Pairwise *F*_ST_ values analyzed for cluster pairs were significant for all pairs of clusters (Table 5).

**Table 5:** Pairwise *F*_ST_ values and respective *Nm* values in three clusters

	Cluster I	Cluster II	Cluster III
Cluster I	0	1.76	1.72
Cluster II	0.14230	0	2.80
Cluster III	0.14540	0.08917	0

Below diagonal – *F*_ST_ values, above diagonal – *Nm* values, *p* value – 0.0000 for all pairs

## Discussion

This is the first study to describe the population genetic structure of *An. culicifacies* sibling species E in the complex that consists of three sympatric clusters in Sri Lanka. The microsatellite markers used in this study were isolated from sibling species A in India [19]. Among the eight markers that were successfully amplified for species E in Sri Lanka, five (AcAVIIIB40, AcA59, AcAVIB213, AcAIIB5, AcAVB93 and AcAVB93A) were highly polymorphic, and thus useful for exploring the genetic population structure of sibling E in Sri Lanka. These five loci had high allele diversity and expected heterozygosity (>0.60) that resulted in the observed genetic diversity of the study population.

Out of the eight microsatellite loci used to screen the sibling species E in the current study, six were included in the set of loci used for sibling species A in India (AcAVIIB40, AcA59, AcAVIB213, AcA11B5, AcAVB93 and AcAVB93A). The number of alleles seen in this study varied according to the microsatellite loci studied. Locus AcA59 had the highest number of alleles (14) compared to 8 alleles for the same locus of species A in India. Meanwhile, locus AcAVIB213 had 12 alleles, while the Indian sibling species A had 17. The fewest alleles were seen for locus AcAVB93A in both Sri Lankan E and Indian A [20]. Furthermore, the allele sizes observed in this study for sibling E were lower than that for sibling A [19] in India for five microsatellite loci (AcAVIIIB40, AcA59, AcAVIB213, AcA75, AcAVB93A), while the sizes of the other three loci (AcAVB93; AcA36; AcAIIB5) of species A in India were within the ranges observed for sibling E in Sri Lanka.

The genetic structure of a sibling species in the *An. culicifacies* complex, given that a study of spatial or Bayesian genetic structure has not been undertaken for any sibling species in the complex. These microsatellite loci have not been physically mapped to *An. culicifacies* polytene chromosomes, and therefore the location of these loci with respect to the polymorphic chromosome forms is unknown. Linkage disequilibrium was detected only within two pairs of loci in the entire population out of ten comparisons as well as in a single pair of loci at all collecting sites, suggesting the absence of significant linkage between loci, which may have a random distribution in the genome.

Samples isolated from Kataragama site agreed with HWE with excess heterozygosity while all other localities showed significant deviations. The heterozygote deficiency observed at all other sites could be due to the population substructure (Wahlund effect), inbreeding, selection or null alleles. The heterozygote deficiency observed at multiple loci likely was not due to selection, which generally engages only one locus [14]. If inbreeding occurs in the population, heterozygote deficiency would be observed at all loci in a population since inbreeding has a genome-wide effect. The heterozygote deficiency could instead be due to null alleles as result of nucleotide mismatches in the primer annealing regions that lead to non-amplification of corresponding alleles. The population substructure analysis revealed three sympatric clusters in the studied population, but Kataragama was the only location that had excess heterozygosity, consisting of only two clusters while all other sites had a mixture of all three clusters.

In this study a possible barrier to gene flow was observed in the East-west direction across the central area of the country that was close to the Kandy sampling site, which has one of Sri Lanka’s highest altitudes and is surrounded by a number of hills (Fig. 1). These hills could act as a barrier to gene flow. Furthermore, Anuradhapura and Nikaweratiya are low-altitude areas on one side of the barrier where cluster I predominated (Fig. 3), while Kataragama, Monaragala and Thanamalwila are situated at a low altitude on the other side of the barrier where cluster III was more common. Interestingly, the Kandy population had nearly equal proportions of each cluster.

The Kataragama site has the lowest altitude and (Fig. 1) is near the sea and has the driest climate. The other sites were closer to the mountain ranges rather than to the ocean. Therefore, the significant variation in the genetic analysis of *An. culicifacies* E for the Kataragama site relative to the other collection sites could arise from influences by the coastal environment.

All sample collecting sites had similar topologies as far as breeding sites for *An. culicifacies* E are concerned, although environmental factors such as temperature and humidity might vary among these sites. Therefore, the availability of breeding sites was not expected to influence the gene flow between collection sites. Most of the areas that *An. culicifacies* inhabits experience high amounts of rainfall only in the South West Monsoon season (December to February). During this period, the abundance of mosquitoes falls drastically due to monsoon-driven flushing of egg clutches and larval breeding sites. Thus, mosquito population abundance undergoes seasonal changes with high densities reported only during the dry seasons. The high level of genetic diversity observed in this study suggests that *An. culicifacies* E can maintain a relatively high effective population size despite large population fluctuations.

Finally, studying insecticide susceptibility and performing parasite susceptibility tests to determine insecticide resistance levels and vectorial capacity of species E in two regions separated by a physical barrier is recommended to provide baseline genetic information about the vector. Such knowledge is useful for implementing new vector control strategies or to revise ongoing national malaria control practices, as well as to drive a re-emergence of malaria prevention programs.

## Conclusions

Three sympatric clusters were detected among *An. culicifacies* E specimens collected in Sri Lanka. There was no effect of geographic distance on genetic differentiation in a pairwise population analysis. The central mountain ranges in Sri Lanka appeared to act as a barrier to gene flow.
